# Xenograft bone-patellar tendon-bone ACL reconstruction: a case series at 20-year follow-up as proof of principle

**DOI:** 10.1186/s40634-023-00651-7

**Published:** 2023-09-06

**Authors:** Kevin R. Stone, Ann W. Walgenbach, Thomas J. Turek, John V. Crues, Uri Galili

**Affiliations:** 1 The Stone Clinic, San Francisco, CA USA; 2 Stone Research Foundation, San Francisco, CA 94123 USA; 3 Radnet, Los Angeles, CA USA; 4 Division of Cardiology, Department of Medicine, Rush University Medical College, Chicago, IL USA

**Keywords:** Anterior cruciate ligament, Xenograft, Knee, Case series

## Abstract

**Purpose:**

ACL reconstruction has a significant failure rate. To address the need for inexpensive strong tissue, a treatment process to “humanize” porcine tissue was developed and tested in primates and humans. This report describes the long-term outcomes from the first human clinical trial using a porcine xenograft ACL reconstruction device.

**Methods:**

The study was performed with Z-Lig™ xenograft ACL device in 2003 as a pilot clinical feasibility study. This device was processed to slow its immune-mediated destruction by enzymatic elimination of α-gal epitopes and by partial crosslinking to slow the infiltration of macrophages into the biotransplant.

**Results:**

Ten patients underwent reconstruction with the Z-Lig™ device. Five of 10 patients failed due to subsequent trauma (*n* = 3), arthrofibrosis (*n* = 1), and surgical technical error (*n* = 1). One patient was lost to follow-up after the 12-year evaluation. Each remaining patient reported a stable fully athletic knee. Physical exams are consistent with a score of less than one on the ACL stability tests. MRIs demonstrate mature remodeling of the device. There is no significant degradation in patient-reported outcome scores, physical exams, or MRI appearance from 12 to 20-year follow-ups.

**Conclusions:**

The studies in a small group of patients have demonstrated that implantation of porcine ligament bioprosthesis into patients with torn ACLs can result in the reconstruction of the bioprosthesis into autologous ACL that remains successful over 20 years. The possibility of humanizing porcine tissue opens the door to unlimited clinical material for tissue reconstructions if supported by additional clinical trials.

**Level of evidence:**

IV, case series.

## Introduction

The ACL is a crucial stabilizer of the knee joint and is frequently damaged during athletic activities. The current surgical method for reconstructing torn ACLs involves the grafting of either autologous or allogeneic (i.e., cadaveric) tendons. Although both methods are successful, they have disadvantages and risks. Harvest of an autograft patellar tendon requires a second surgical site, thereby increasing longer recovery periods, pain, and morbidity, whereas many allogeneic tendons are of elderly individuals with low biomechanical capacity and may cause transmission of hepatitis and other infectious diseases [13, 15]. Therefore, there is a need to explore alternative methods. Previous pre-clinical research has investigated a porcine bone-patellar tendon-bone bioprosthesis that has been processed to reduce rejection by enzymatic elimination of α-gal epitopes from the implanted bioprosthesis and partial crosslinked with glutaraldehyde, to determine if it can successfully be used in humans as a viable ACL reconstruction [10, 18–20]. Based on these pre-clinical studies we hypothesized that enzymatic elimination of α-gal epitopes prevents subsequent accelerated destruction of implanted tissues by the natural anti-Gal antibody, and the partial crosslinking by glutaraldehyde molecules results in their function as “speed bumps” that slow the infiltration of macrophages into the biotransplant. The combination of these two treatments slows the immune-mediated destruction of the porcine bioprosthesis, thereby enabling the gradual reconstruction of the bioprosthesis into viable autologous ACL that functions for many years in the treated patient. Since the processing of porcine ligament bioprosthesis for its in situ remodeling into autologous functional ACL was never demonstrated, the clinical question was whether a porcine orthopaedic bioprosthesis can be processed in a way that it can undergo reconstruction into an autologous live human ACL that can function indefinitely. A pilot clinical study was undertaken as a potential proof of principle to test a porcine bone-patellar tendon-bone ACL replacement device for years (Z-Lig™^)^) in ten human patients requiring ACL reconstruction [17]. The study was approved by the FDA under an Investigational Device Exemption as a pilot clinical feasibility investigation. The study protocol was approved by an institutional review board and all patients entered the study after informed consent. The device was prepared under controlled manufacturing conditions using the same methods as preclinical materials. All subjects received the porcine xenograft device. The objective of the study was to validate the safety, implantability, and technical feasibility of the xenograft device. We report on the long-term efficacy at the 12- and 20-year time points.

## Patients and methods

### Study design

The ten patients included in this case series underwent reconstruction with the Z-Lig™ xenograft ACL device and were prospectively followed over time until device failure or loss to follow-up. Porcine bone-patellar tendon-bone grafts were processed prior to implantation using recombinant alpha-galactosidase enzyme, low-level glutaraldehyde and terminally sterilized with 17.8 kGy irradiation [17, 20]. This processing was based on preclinical studies indicating that enzymatic elimination of α-gal epitopes prevents subsequent accelerated destruction of implanted tissues by the natural anti-Gal antibody, whereas the partial crosslinking by glutaraldehyde molecules results in their function as “speed bumps” that slow the infiltration of macrophages. All patients received the porcine xenograft. All grafts were harvested and sized in-house to match 10 mm by 25 mm bone blocks with 10 mm diameter grafts. All ACLs were placed with an outside-in two-incision technique with the same interference screw fixation on the femur and tibia. The patients had a mean age of 41 years (21 to 51 years), with a distribution of seven males and three females. All patients were enrolled under IRB approval and consent from the primary author’s private practice. The patient population was extremely athletic, presenting with an average incoming Tegner Activity Score of 8.0 (6.0 to 10.0) which indicates the ability to perform high-level competitive sports. Fifty percent presented with complex chronic etiologies (injury to surgery > 3 months). Previous surgeries to the affected/operative knees included: two ACL revisions; one ACL repair and three medial meniscal repairs. Three patients had previous ACL surgery of the contralateral knee.

### Primary study endpoints

Endpoints were effusion and knee stability as assessed via clinical examination by the principal investigator and an independent orthopaedic surgeon in the first two years and then by the PI at 12 and 20 years post-operation. Knee laxity assessments included: Instrumented KT-1000 [16], Pivot Shift, Anterior Drawer [2], and Lachman tests [22]. Supplemental evaluations included antibody titers for anti-Gal and anti-non-Gal and evaluation of graft integrity and maturation by MRI.

### Secondary endpoints

Secondary analysis of the clinical investigation involved subjective evaluations through standardized reporting tools and included: improvement in activity level as measured by Tegner Activity Scale [21]; improvement in function and pain as measured by IKDC subjective patient questionnaire [1]; and improvement in quality of life as measured by SF-36 [23].

### Statistical analysis

Statistical inferences were made on clinical outcomes of objective evaluations for the KT-1000 Manual Max, Lachman scores, Anterior Drawer scores, and Pivot Shift scores as well as for the subjective evaluations of International Knee Documentation Committee (IKDC) questionnaire, SF-36, and Tegner activity instruments. Changes from baseline scores were analyzed using paired t-tests and a 1-sided alpha level of 0.05. No adjustments for multiple timepoint comparisons were made.

This report covers long-term follow-up of evaluable Z-Lig™ implanted subjects comparing the mid-term results to the long-term (20-year outcomes) with knee laxity, subjective evaluations, and MRI assessment.

## Results

### Device implantability and safety

The device and surgery were well tolerated by all patients. Intra-operative surgical and technical feasibility objectives were met with device handling comparable to human patellar-tendon allografts. Five of six evaluable subjects presented with functional grafts at the 12-year post-operative time point and satisfied all study success criteria including effusion, KT-1000, Pivot Shift, Lachman, and Anterior Drawer tests. Early transient effusion was noted in three subjects which were resolved by 12 months. One of these six assessable subjects presented with tibial bone plug loosening at 15 months after Z-Lig™ reconstruction, then underwent device removal and cancellous allografting to the tibial tunnel. Four non-evaluable subjects of the ten total enrolled in the study had non-device related adverse effects during the study as follows: two traumatic reinjuries at 3.5 and 8 months postoperatively, one failure due to arthrofibrosis, and one impingement technical/surgical complication. Re-ruptures and problems encountered in these four subjects reflect the aggressive return to sports and complex knee etiologies present in this study population and were not deemed to be related to the Z-Lig™ device by the independent review panel. No adverse events occurred in the 2–20 year interval for the remaining four assessable subjects.

### Serology and clinical chemistries

**Fig. 1:**
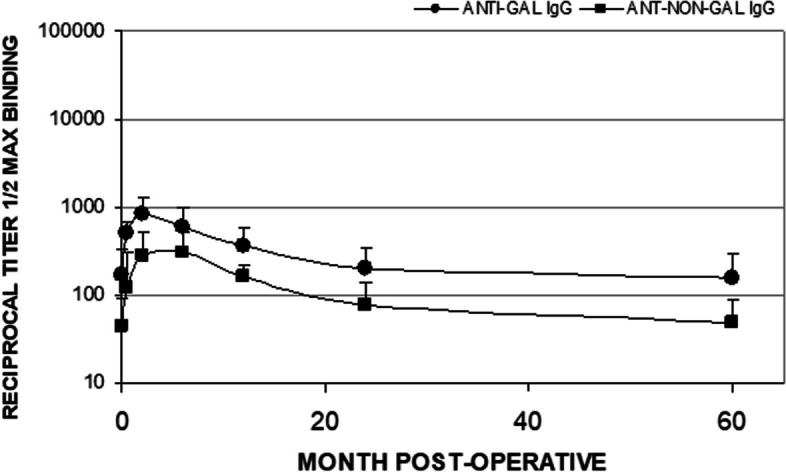
Average anti-Gal IgG and anti-non-Gal IgG titers for *n* = 5 subjects through 60 months are presented as mean plus standard deviation

Antibody response to the graft was consistent with preclinical models and showed a slight increase in the anti-Gal IgG antibody at two months followed by resolution to the pre-implantation range in the 6 to 24-month interval. Antibodies to other pig antigens (anti-non-Gal IgG) peaked at 6 months and declined in the 12 to 24-month interval in all patients. Both anti-Gal and anti-non-Gal titers were comparable to baseline at 24 and 60-month intervals, suggesting that it took ~ 2 years to eliminate the porcine tissue antigens to convert the pig ligament tissue into human autologous ACL tissue. Concurrent monitoring of anti-Gal and anti-non-Gal IgM titers showed only marginal increases. No adverse clinical response could be attributed to serological findings. The resolution of anti-non-Gal titers correlates with the active remodeling of the porcine graft and replacement by host tissue. These were not repeated at the 12 and 20-year time intervals. Average anti-Gal and anti-non-Gal IgG titers for evaluable patients are shown in Fig. 1.

Blood chemistry, urine chemistry, and serology results were within acceptable normal ranges except as discussed below. C-Reactive Protein (6 subjects) and sedimentation rate (3 subjects) lab values were transiently elevated between the two- and eight-month time period signaling a resolved inflammatory response. White blood cell differential counts were slightly elevated in three subjects (eosinophilic 2 subjects; lymphocytic 1 subject) between 2 and 12 months. Laboratory values were within normal ranges in the 12 to 24-month time interval. Blood chemistry and urine analysis were not performed after the 24-month time point.

### Clinical outcomes

**Fig. 2:**
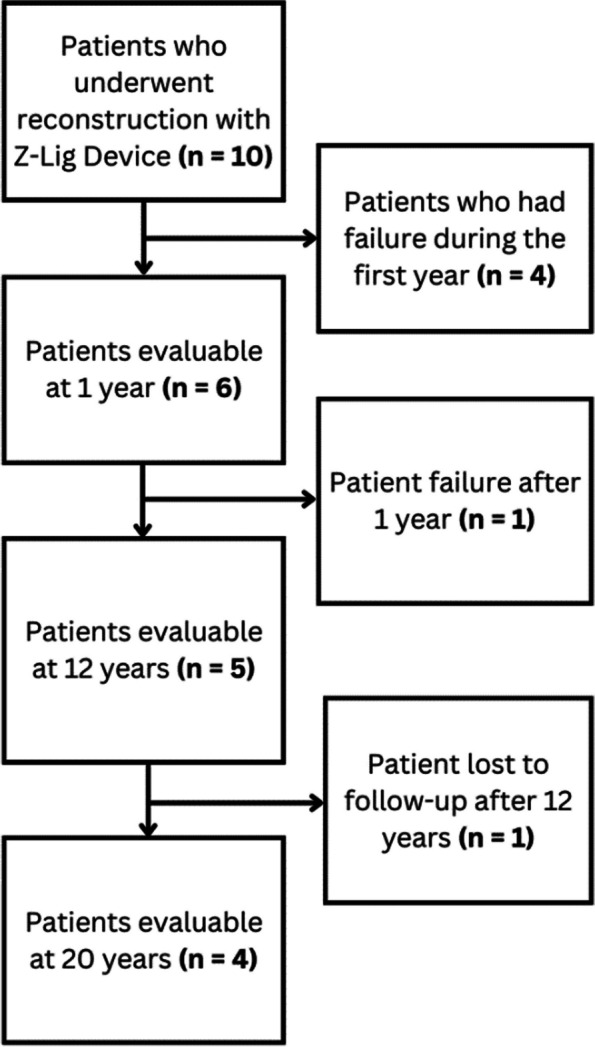
Flowchart of the ten patients who underwent reconstruction with the Z-Lig™ xenograft ACL device

**Fig. 3:**
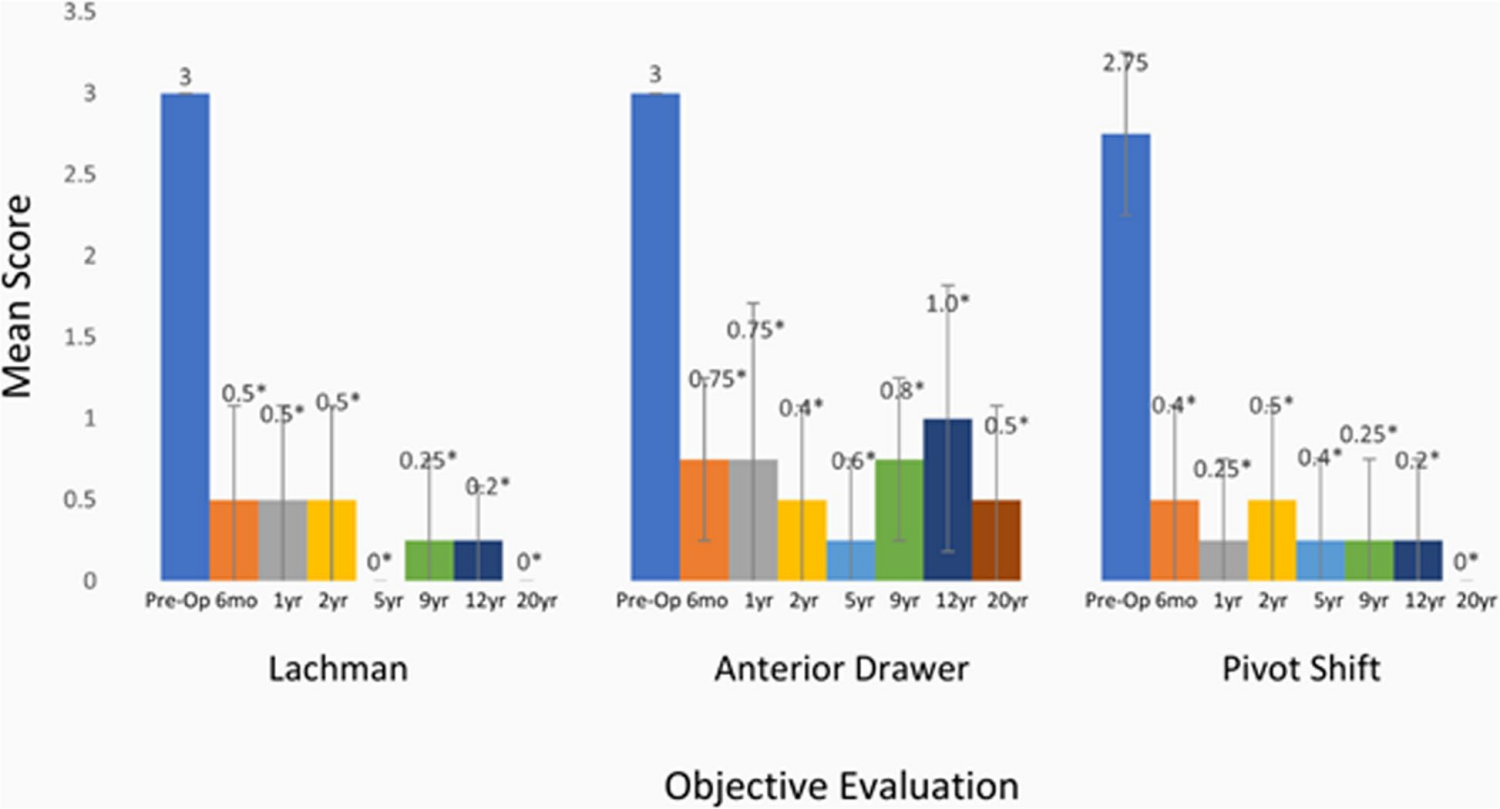
Average knee laxity assessments for the four evaluable subjects (*n* = 4). Data are presented as mean plus standard deviation. * *p* < 0.05 for comparison of pre-operative results to either 6 months, or 1, 2, 5, 9, 12, or 20 years post-operative results

The final population of the case series was composed of four patients as presented in Fig. 2 showing the study flow over time. Primary outcome evaluations were conducted by both the principal investigator and independent orthopaedic surgeon to maintain objectivity in the study in the first two years and then by the PI at 12 and 20 years postoperatively. In the 12-year and 20-year evaluable subjects, all stability assessments demonstrated significant improvement compared to pre-operative assessments over the course of the study. Evaluations for KT-1000 Manual Max were only available for the 5 evaluable subjects at the 12-year follow up and scores improved from 5.25 to 1.4 (*p* = 0.03). The following summarizes the results for the final 4 evaluable subjects at 20 years: Lachman scores improved from 3.0 to 0.25 (*p* < 0.001); Anterior Drawer scores improved from 3.0 to 1.0 (*p* = 0.008) and Pivot Shift scores improved from 2.75 to 0.25 (*p* = 0.002). Figure 3 shows the mean knee stability assessment data for the four evaluable subjects: P-01, P-03, P-04, and P-07.

**Fig. 4:**
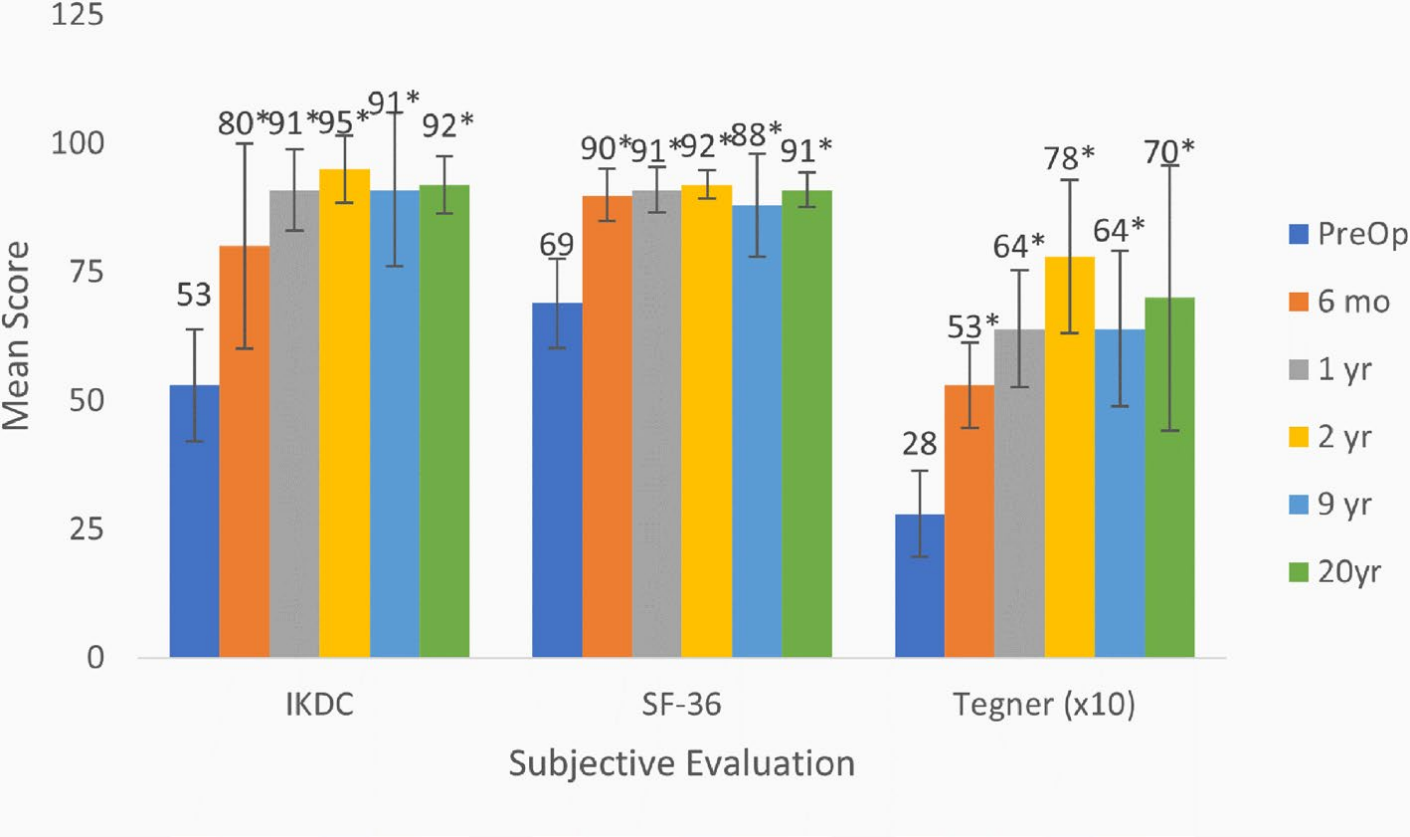
Average subjective evaluations for the four evaluable subjects (*n* = 4). Data are presented as mean plus standard deviation. * *p* < 0.05 for comparison of pre-operative results to either 6 months, or 1, 2, 5, 9, 12, or 20 years post-operative results

The evaluable subjects were also assessed for improvement in subjective measures with 5 patients at 12 years and 4 patients at 20 years. Subjective outcome measures of subject self-assessment also improved when comparing pre-operative and 12-year (and 20-year outcomes) outcomes: Tegner Activity: 2.8 to 6.8 (*p* < 0.001); IKDC Subjective: 49 to 94 (*p* < 0.001) and SF-36: 68 to 91 (*p* = 0.015). The average values are for the four assessable subjects: P-01, P-03, P-04, and P-07. Subjective evaluations are presented in Fig. 4.

### MRI assessment

**Fig. 5:**
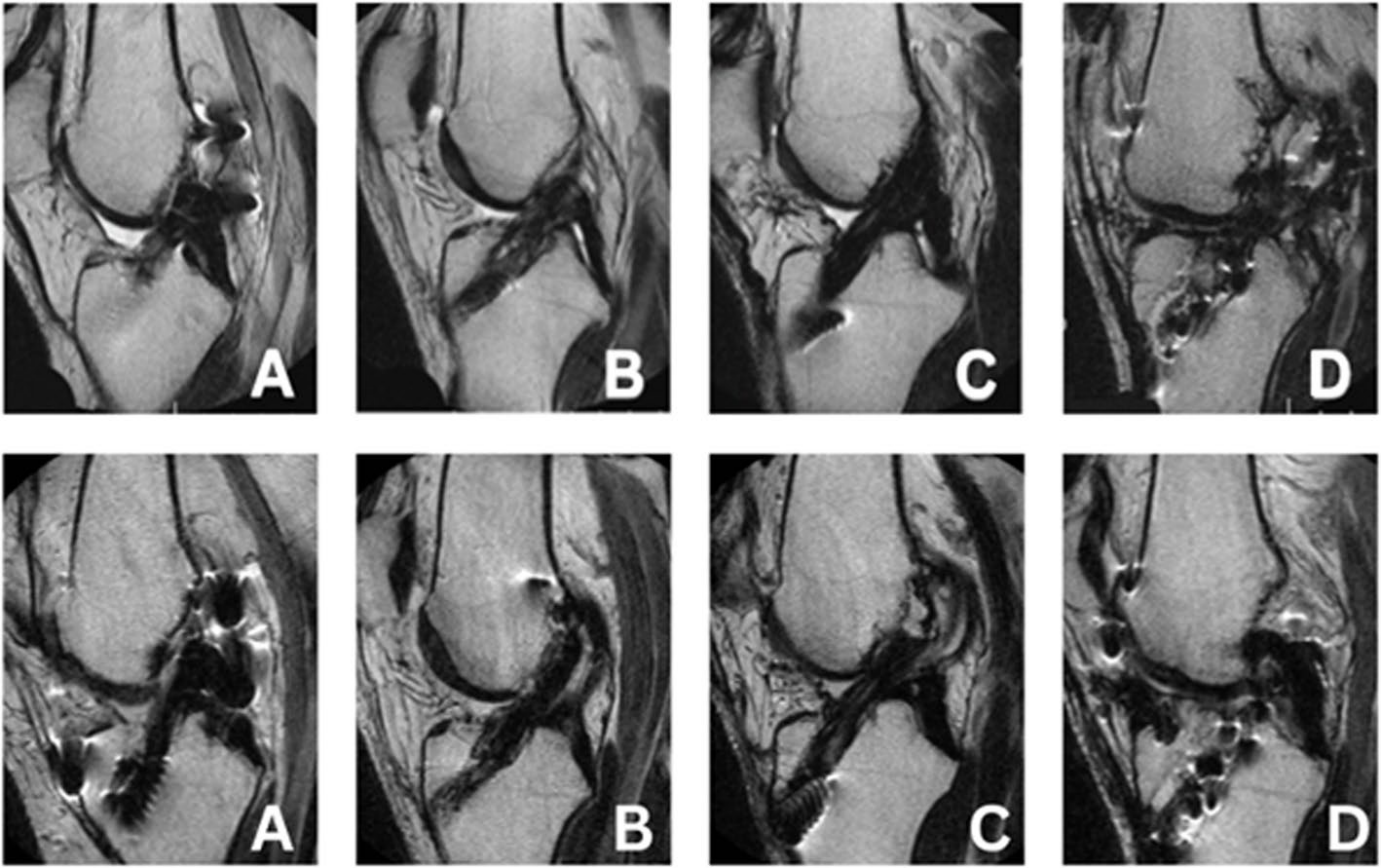
12-year (top row) and 20-year (bottom row) MRI Images of Z-Lig™ Reconstructions, FSE T-2 weighted in the sagittal plane: **A**) Subject P-01; **B**) Subject P-03; **C**) Subject P-04; and **D**) Subject P-07

MRI evaluation was performed on operative knees on a 1.0 Tesla ONI extremity scanner. Five data acquisition series were obtained: 1) coronal plane with T1 weighting; 2) coronal plane with STIR technique; 3) axial plane with T2 weighting; 4) sagittal plane with proton density technique; and 5) sagittal plane with T2 weighting. Table 1 summarizes radiology report comments and graft status at the 12-year follow-up. Parallel T-2 weighted sagittal images of the Z-Lig™ reconstructed ACL are shown in Fig. 5A-D. Subject 7 presents with a titanium particulate artifact attributed to revision screw removal. Table 2 summarizes radiology report comments and graft status at the 20-year follow-up.

**Table 1:** MRI Assessment: 12 years post-operative

**SUBJECT**	**RADIOLOGIST COMMENTS**	STATUS
P-01	The anterior cruciate ligament is intact with no evidence of tearing. The posterior cruciate ligament is intact. The medial and lateral menisci demonstrate normal signal characteristics and morphology (Fig. 5A)	Intact
P-03	The anterior cruciate ligament graft itself is intact without evidence of degeneration or surrounding inflammation. It is unchanged in appearance as compared to the prior MRI evaluation. The posterior cruciate ligament is unremarkable (Fig. 5B)	Intact
P-04	The posterior cruciate ligament is intact. An anterior cruciate ligament is noted in place. The graft fibers demonstrate a mildly increased signal that has improved since the prior examination and are intact (Fig. 5C)	Intact
P-07	The anterior cruciate ligament is unchanged in appearance with a persistent tear at the proximal attachment and adhesive scarring of the distal graft to the posterior cruciate ligament. The posterior cruciate ligament graft is intact (Fig. 5D)	Intact
P-10	The anterior cruciate ligament graft is intact with normal signal intensity. The posterior cruciate ligament is intact	Intact

**Table 2:** MRI assessment: 20 years post-operative

SUBJECT	RADIOLOGIST COMMENTS	ACL STATUS
P-01	The ACL graft fibers are intact. The posterior cruciate ligament is intact. Horizontal tear of the posterior horn and body of the lateral meniscus with a small peripheral body extrusion (Fig. 5A.)	Stable
P-03	Anterior cruciate ligament reconstruction with an intact graft. Femoral tunnel in the 11 o'clock position in the coronal plane and tibial tunnel in the middle third of the tibial plateau. The moderately blunted appearance of the posterior horn and body of the medial meniscus is consistent with prior partial meniscectomy (Fig. 5B)	Stable
P-04	Anterior cruciate ligament reconstruction is intact without a tear. Mild remote injury of the proximal medial collateral ligament. Blunting of the medial meniscus body free edge is consistent with prior partial meniscectomy (Fig. 5C)	Stable
P-07	Anterior cruciate ligament reconstruction is unchanged. Postsurgical changes of medial meniscal allograft transplantation [17 years post-Z Lig™ implantation] with grade 2 signal noted in the posterior horn but otherwise intact graft (Fig. 5D)	Stable
P-10	Lost to follow-up	

## Discussion and conclusions

This study was performed to determine whether a proof of principle can be achieved regarding the possibility of processing porcine orthopaedic bioprostheses of bone-tendon-bone in a way that can result in the reconstruction of such bioprostheses into autologous live human ACL that can function indefinitely implanted patients. For this purpose, the porcine tissue was enzymatically depleted of α-gal epitopes and partially crosslinked by glutaraldehyde. These treatments resulted in slowing the infiltration rate of macrophages destroying the implanted porcine tissue. This enabled the gradual infiltration of the patient’s fibroblasts to infiltrate into the porcine implant, align with the porcine collagen fibers matrix and secrete their autologous collagen fibers. Patients with ruptured ACL were implanted with these porcine bioprostheses and the reconstruction of the autologous functional ACL was monitored for 20 years. Five of six evaluable subjects receiving the Z-Lig™ device are categorized as successful ACL reconstructions and met all primary and secondary criteria for functional knee outcome at 12 years and four patients (one lost) at 20 years postoperatively. Twelve-year assessments confirmed the 9-year findings and the 20-year evaluations confirmed the 12-year findings. MRI results for three of the four available patients who underwent 20-year Z-Lig™ reconstructions indicated primarily contiguous grafts with maturation and normalizing signal density. The fourth patient exhibited a persistent tear at the proximal attachment at both 12-year and 20-year follow-ups. The long-term functional efficacy, MRI evaluation, and normalized serology further support viable attenuation of immunological recognition and functional host ligamentization of the xenograft Z-Lig™ device.

According to our hypothesis, the gradual reconstruction of the porcine tissue with autologous human tissue within a period of 1–2 years could be demonstrated in the explanted bioprostheses of non-evaluable patients. Histopathology of the explanted porcine tissue in these failed patients demonstrated neo-vascularization of the implant, gradual destruction of the pig ligament tissue by infiltrating macrophages, and its reconstruction by human fibroblasts that align with the porcine collagen fibers and producing their own collagen fibers which replace the porcine collagen scaffold [17].

The major stumbling block for the transplantation of animal tissues into humans has been acute immunological rejection. The primary cause of this rejection has been identified as a cell and matrix surface carbohydrate antigen called the α-galactosyl epitope [7, 8]. Humans and Old-World primates lack the α-gal epitope, but all other mammals produce and incorporate α-gal epitopes into cellular and extracellular structures using the α-1,3galactosyl transferase enzyme [9, 11]. Humans and Old-World primates continuously produce anti-Gal antibodies constituting about 1% of circulating immunoglobulins [9] and are therefore not immunotolerant towards exogenous α-gal. Consequently, transplantation of xenogeneic tissue presenting α-gal epitopes in Humans and Old-World primates results in host recognition and acute rejection [4, 5, 7, 8].

Xenograft tissues that are tested in animal-to-animal studies fail to demonstrate the anti-Gal response seen when animal tissues are implanted into Old-World primates or humans [8, 15]. The novelty of the tissue treatments described in this study is primarily the use of the α-galactosidase enzyme to remove the key carbohydrate responsible for 95% of the rejection phenomenon when animal tissues are transplanted into humans [18]. All other efforts to modulate xenograft tissues have resulted in acute or chronic rejection if live tissues were used [4] and inflammation and fibrosis if glutaraldehyde fixed tissues or tissues when only cellular depletion was employed [3, 6, 14]. The elimination of these α-gal epitopes permits “humanization” of the xenograft tissue and the remodeling of transplanted tendons into ligaments, a process called ligamentization [12, 17]. In this process macrophages infiltrating the porcine tissue gradually destroy it. Human fibroblasts follow these macrophages, align with the porcine collagen fibers, secrete their collagen fibers, and gradually replace the porcine tissue with autologous ACL. Concomitantly, the porcine bone blocks are remodeled into integrated human bone tissue. These processes take approximately two years; during that period, the xenografts maintain their biomechanical efficacy. These xenograft bone-patellar tendon-bone grafts ligamentized over time and removed the remaining pig antigens, demonstrating efficacy and successful MRI appearance. This bodes well for further study and applications of enzyme-treated animal tissues in humans.

A primary limitation of this study is the small sample size typical of a pilot study for safety and the inclusion of very complicated knees in high-activity patients. At the time of patient selection, the thinking was to choose the most difficult cases to learn from rather than selecting low-activity pristine knees. From a product success point of view, this was a mistake. A less aggressive rehabilitation schedule with compliance monitoring should have been incorporated. It is also unknown whether an even stronger graft would have prevented the two early traumatic failures. While not reported in this pilot study, the marked diminishment of pain and disability when compared to autogenous harvesting of the patient's own patellar tendon is a proposed benefit of xenograft donor tissues.

## Conclusions

In summary, the Z-Lig™ pilot study objectives of safety, technical feasibility, and implantability were met along with encouraging long-term data through twenty years in four of the patients studied. The reconstruction period of the porcine tendon bioprosthesis into autologous human ACL is ~ 2 years. To our knowledge, this is the first study demonstrating the in situ conversion of a processed and fix porcine tissue into a viable and functional human tissue. The possibility of humanizing animal tissue opens the door to unlimited clinical ligamentous and fibrous tissues if supported by additional clinical trials.

## Data Availability

The datasets generated during the current study are not publicly available but are available from the corresponding author upon reasonable request.
